# Metabolomics analysis reveals amelioration effects of yellowhorn tea extract on hyperlipidemia, inflammation, and oxidative stress in high-fat diet-fed mice

**DOI:** 10.3389/fnut.2023.1087256

**Published:** 2023-01-19

**Authors:** Na Ta, Lisha A., Erdunduleng E., Rigeer Qi, Xiyele Mu, Lan Feng, Genna Ba, Yonghui Li, Junqing Zhang, Laxinamujila Bai, Minghai Fu

**Affiliations:** ^1^Key Laboratory of Tropical Translational Medicine of Ministry of Education, Hainan Provincial Key Laboratory for Research and Development of Tropical Herbs, School of Pharmacy, Hainan Medical University, Haikou, China; ^2^NMPA Key Laboratory for Quality Control of Traditional Chinese Medicine (Mongolian Medicine), School of Mongolian Medicine, Inner Mongolia Minzu University, Tongliao, China; ^3^Department of Mongolian Medicine Preparation, The Affiliated Hospital of Inner Mongolia Minzu University, Tongliao, China

**Keywords:** yellowhorn tea extract, hyperlipidemia, inflammatory factor, oxidative stress, liver untargeted metabolomics

## Abstract

Yellowhorn tea (YT) is traditionally used as a lipid-lowering beverage in Mongolian minorities. However, the pharmacological effects of YT extract and its specific metabolic changes in hyperlipidemia models are not fully understood. The aim of this study was to identify biomarkers using untargeted metabolomics techniques and to investigate the mechanisms underlying the changes in metabolic pathways associated with lipid lowering, anti-inflammation and anti-oxidant in hyperlipidemic mice. A high-fat diet (HFD)-induced hyperlipidemic mouse model was established. YT extract was administered as oral gavage at 0.15, 0.3, and 0.6 g/kg doses for 10 weeks. HFD-induced hyperlipidemia and the therapeutic effect of YT extract were evaluated based on histopathology and by assessing blood lipid levels. Liver inflammatory factors and oxidative stress indices were determined using enzyme-linked immunosorbent assays. Liver metabolites were evaluated using untargeted metabolomics. Biochemical and histological examinations showed that YT extract significantly reduced body-weight gain (*p* < 0.01) and fat deposition in tissues. YT extract significantly reduced the levels of serum and liver triglyceride and total cholesterol; inflammatory factors [interleukin (IL)-6, IL-1β, and tumor necrosis factor-α]; malondialdehyde; and leptin (*p* < 0.05) in hyperlipidemic mice. YT extract also significantly increased the levels of oxidative stress indicators (superoxide dismutase, catalase, and glutathione peroxidase) and adiponectin. Metabolomics studies revealed several endogenous molecules were altered by the high-fat diet and recovery following intervention with YT extract. The metabolites that were significantly different in the liver after YT intake included citicoline, acetylcholine, pyridoxine, and NAD. Pathway analysis indicated that YT extract ameliorated HFD-induced hyperlipidemia in mice via three major metabolic pathways, namely, glycerophospholipid metabolism, vitamin B6 metabolism, and nicotinate and nicotinamide metabolism. This study demonstrates YT extract has profound effects on the alleviation of HFD-induced hyperlipidemia, inflammation and oxidative stress.

## Introduction

Hyperlipidemia is a pathological state of metabolic disorder characterized by an abnormal elevation of plasma total cholesterol (TC), triglyceride (TG), low-density lipoprotein-cholesterol (LDL-C) levels, and an abnormal decrease in high-density lipoprotein-cholesterol (HDL-C) level (1). The causes of hyperlipidemia are diverse and include unhealthy eating habits, psychology, diabetes, pathology, and genetics (2–4). Hyperlipidemia is a major contributor to numerous cardiovascular diseases such as essential hypertension, coronary heart disease, atherosclerosis, and diabetes (5, 6). Previous studies have reported that treatment of hyperlipidemia can reduce the mortality rate of cardiovascular diseases. In China, the prevalence of HLP in adults is 18.36%, which is >160 million (7). The excessive intake of high-fat diet (HFD) leads to an increase in plasma cholesterol levels, thereby posing a serious threat to cardiovascular health in humans (8). Studies have shown that chronic and excessive intake of HFD leads to adipose deposition in visceral organs (perirenal and epididymal adipose tissue), increasing chronic inflammation and leading to damage due to oxidative stress (9–11). Therefore, inhibition of chronic inflammation and oxidative stress damage could be an effective strategy to reduce the risk of metabolic diseases. Pharmacological interventions are the primary treatment for hyperlipidemia; however, most drugs can cause adverse reactions such as abdominal discomfort, bloating, and diarrhea (12). In particular, the long-term use of statins is associated with adverse effects such as muscle toxicity and liver damage (13). Traditional Chinese Medicine (TCM) has been increasingly investigated for their efficacy in treating hyperlipidemia. The lipid-lowering mechanisms of TCM are possibly summarized as: (1) inhibiting intestinal lipids absorption; (2) lowering endogenous cholesterol production; (3) modulating cholesterol transport; (4) promoting the liver cholesterol excretion; (5) modulating lipid-related transcription factors (14–16). The use of natural medicine may be a suitable approach in alleviating dyslipidemia and avoiding adverse reactions.

*Xanthoceras sorbifolium* Bunge is a TCM herb that has a long history of medicinal use. It is the only species of the genus *Xanthoceras* belonging to the Sapindaceae family (17), and is a native plant in northwestern China that is mainly distributed in Shaanxi, Shanxi, Hebei, Gansu, and Inner Mongolia (18, 19). Modern pharmacological studies have shown that its active ingredients are primarily triterpenoids, flavonoids, phenolic acids, and saponins, which have a wide range of pharmacological activities, including antioxidant, antineuritic, antitumor, antilipogenesis, anti-obesity, anti-HIV, gastric protection, immunomodulatory, and anti-inflammatory activities (20). The tender leaves of *X. sorbifolium* can be used as raw material to prepare tea that has health-promoting benefits (21). Studies have reported that the long-term consumption of yellowhorn tea (YT) has effects of diuretic hemostasis, dispelling dampness, and lowering blood lipids and cholesterol (22–28). However, to the best of our knowledge, none of the studies have reported the antihyperlipidemic effect of YT. Accordingly, we conducted an *in vivo* study to determine the antihyperlipidemic effects of YT.

Metabolomic studies are used to systematically identify and quantify the levels of metabolites, and to elucidate the pathogenesis of diseases and the mechanism of action of drugs (29). Currently, metabolomics is used for the diagnosis of hyperlipidemia, drug therapy monitoring, drug development, and in studies related to food and nutrition science, among other fields (30–32). Liquid chromatography–mass spectrometry (LC-MS) is the most widely used tool in metabolomics (33, 34), which was used in our study too, owing to its suitability for our research (35). To date, metabolomics has not been used to evaluate the effects of YT extract on dyslipidemic mice. In this study, serum biochemical analysis, enzyme-linked immunosorbent assay (ELISA)-based determination of hepatic enzymes, histopathological analysis, and metabolic pathway analysis were performed, and metabolic changes were analyzed to determine the lipid-lowering, anti-inflammatory, and antioxidant mechanisms of YT on HFD diet-induced hyperlipidemic mice.

## Materials and methods

### Chemicals and reagents

Yellowhorn tea was harvested in Kezuo Middle Banner, Tongliao, Inner Mongolia, China. HFD was purchased from Changchun Yi Si Experimental Animal Technology Co., Ltd. (No.: SCXK [Lu] 2021 0003, Changchun, China). Simvastatin was purchased from Shanghai Xinyi Pharmaceutical Co., Ltd (Shanghai, China). Kits to determine TG, TC, HDL-C, and LDL-C levels were purchased from Shenzhen Icubio Bio-technology Co., Ltd. (Shenzhen, China). ELISA kits for the determination of tumor necrosis factor (TNF)-α (JM-02415M1), interleukin (IL)-6 (JM-02446M1), IL-1β (JM-02323M1), malondialdehyde (MDA, JM-11347M1), superoxide dismutase (SOD, JM-02672M1), catalase (CAT, JM-03116M1), glutathione peroxidase (GSH-Px, JM-03038M1), leptin (JM-02902M1), and adiponectin (JM-02830M1) were purchased from Jiangsu Jingmei Biotechnology Co., Ltd. (Yancheng, China). Hematoxylin and eosin (H&E) stain was purchased from Nanjing Jiancheng Technology Co., Ltd. (Nanjing, China).

### YT extract preparation and extraction

The tender leaves of YT were collected from the planting base of YT in Tongliao, Inner Mongolia. The harvested young leaves were homogeneous in size and preserved in a pest- and disease-free environment. The leaves were laid out on bamboo mats and left to wilt until the young leaf tips lose their sheen. Kill-green process was conducted by panning in a rolling drum (Shengjie JS-1000, Jining, China) at 220°C for 8 min and followed by rolling the tea leaves into wrinkled strips to make final YT product. Five-hundred grams of YT samples were ground and extracted in boiling water at a ratio of 1:10 for 30 min, after which the fresh tea infusion was filtered through two layers of industrial gauze. The tea dregs were extracted again for 20 min in water (1:8,w/v), and the double-extracted tea infusion was combined and vacuum-filtered. The YT powder was obtained using a spray dryer (Yirui LPG-5, Changzhou, China).

### Chemicals analysis

The compounds of the YT extract were analyzed by LC-MS (Thermo, Ultimate 3000LC, Q Exactive HF). The operating conditions were listed as follows: chromatographic column, C18 column [Zorbax Eclipse C18(1.8 μm × 2.1 × 100 mm)]: column temperature, 30°C; flow rate, 0.3 ml/min; injection volume, 2 μl, Automatic sampler temperature 4°C. Mobile phase A: water/formic acid (99:1, v/v) and mobile phase B: 100% acetonitrile. The gradient program was as follows: 0–2 min, 5% acetonitrile; 2–6 min, 30% acetonitrile; 6–7 min, 30% acetonitrile; 7–12 min, 78% acetonitrile; 12–14 min, 78% acetonitrile; 14–17 min, 95% acetonitrile; 17–20 min, 95% acetonitrile; 20–21 min, 5% acetonitrile; 21–25 min, 5% acetonitrile. Ms conditions: positive and negative mode: heater temperature 325°C; Sheath gas flow rate: 45 arb; Auxiliary gas flow rate: 15 arb; Purge gas flow rate: 1 arb; Electrospray voltage: 3.5 kV; Capillary temperature: 330°C; S-lensrf Level: 55%. Scanning mode: Full Scan (M/Z 100–1,500) and data-dependent mass spectrometry (DD-MS2, TopN = 10); Resolution: 120,000 (primary mass spectrometry) and 60,000 (secondary mass spectrometry). Collision mode: High energy collision dissociation (HCD).

### Animal experiments

Sterile-pathogen-free male Kunming mice (license number SCXK [Liao] 2020-0001) weighing 22 ± 25 g were purchased from Chang Sheng Biotechnology Co., Ltd. (Liaoning, China). All mice were individually housed in a temperature-controlled room at 22–25°C and relative humidity of 30–40% and subjected to a 12 h/12 h light/dark cycle. After 7 days of adaptive feeding, mice were randomly divided into a control group, HFD group, low-dose (0.15 g/kg), medium-dose (0.3 g/kg), or high-dose (0.6 g/kg) YT extract group, or simvastatin (0.01 g/kg) group with six mice per group and labeled. The doses of simvastatin and YT extract administered to mice were determined based on the literature (36). The normal control group was fed a normal diet, whereas the other groups were fed a HFD (crude protein: 24.28%, crude fat: 17.99%, carbohydrate: 71.61%). Mice in the YT extract-treated and positive control groups were fed a HFD and orally administered YT extract and simvastatin, whereas those in the control and model groups were administered an equivalent volume of CMC-Na for 10 weeks simultaneously. Food intake and activity status of mice were observed and recorded daily and mice were weighed every week during the study. After the last dose, mice in each group were fasted; the next day, mice were anesthetized with sodium pentobarbital and blood from the abdominal aorta was collected. Blood samples were stored at room temperature for 30 min and centrifuged at 3,000 rpm for 10 min at 4°C to separate the serum. The heart, liver, kidneys, spleen, and fat tissues were surgically removed from each mouse. The wet weights of organs were recorded and tissues were stored at −80°C until subsequent experiments. All experiments were performed following the protocols reviewed and approved by the Institutional Animal Care and Use Committee, Inner Mongolia Minzu University (approval no. NM-LL-2021-06-15-1).

### Biochemical assays using the serum and liver samples

An automatic biochemical analyzer (Ichem-340, Icubio, Shenzhen, China) was used to measure serum TG, TC, HDL-C, LDL-C levels, and hepatic TG and TC levels. Sample volumes of 150 μl were used for analysis and the absorbance was measured at 450 nm.

### ELISA

Liver homogenates (10%) were prepared by the addition of 100 mg liver tissue to a 0.9% sodium chloride solution (1:9 ratio) and homogenization using an electric tissue grinder in an ice bath. The homogenate was centrifuged at 3,500 rpm for 10 min and the levels of IL-6, IL-1β, TNF-α, SOD, MDA, CAT, GSH-Px, leptin, and adiponectin were determined in the supernatant using the corresponding ELISA kits following the manufacturers’ instructions. A sample volume of 10 μl was used for the assays and the absorbance was measured at 450 nm.

### H&E staining

Adipocyte morphology and the extent of lipid accumulation in the liver were determined using H&E staining. Liver and fat tissues were fixed in 10% formalin and dehydrated overnight using 80% ethanol. Liver sections were embedded in liquid paraffin and sliced into 5-μm-thick sections, whereas fat tissues were sliced into 3-μm-thick sections. The slices were soaked in xylene solution for 30 min twice, soaked in a gradient of ethanol concentrations (95, 90, 80, and 70%), and stained with H&E for 1–2 min.

### Metabolomics analysis of liver samples

Twenty-five milligrams of samples were weighed into an Eppendorf tube and 50 μl of the extraction solution (methanol:acetonitrile:water = 2:2:1, with isotopically labeled internal standard mixture) was added. The samples were homogenized at 35 Hz for 4 min and sonicated for 5 min in an ice-water bath. The homogenization and sonication cycles were carried out three times, after which the samples were incubated for 1 h at −40°C and then centrifuged at 12,000 rpm for 15 min at 4°C. The supernatant was transferred to a fresh glass vial for analysis. Quality control (QC) samples were prepared by mixing an equal aliquot of the supernatants from each of the samples. Liver samples were analyzed for untargeted metabolite profiles using LC-MS/MS (Q Exactive Orbitrap, Thermo Fisher Scientific, Waltham, MA, USA). LC-MS/MS was performed using a UHPLC system (Vanquish, Thermo Fisher Scientific) with a UPLC BEH amide column (2.1 mm × 100 mm, 1.7 μm) coupled to Q Exactive HFX mass spectrometer (Orbitrap MS, Thermo). The mobile phase consisted of 25 mmol/L ammonium acetate and 25 mmol/L ammonium hydroxide in water (pH 9.75) (A) and acetonitrile (B). The auto-sampler temperature was 4°C and the injection volume was 2 μl. The QE HFX mass spectrometer was used to acquire MS/MS spectra in the information-dependent acquisition mode using the acquisition software (Xcalibur, Thermo Fisher). In this mode, the software continuously evaluated the full-scan MS spectrum. The electrospray ionization source conditions were set as follows: sheath gas flow rate, 30 Arb; aux gas flow rate, 25 Arb; capillary temperature, 350°C; full MS resolution, 120,000; MS/MS resolution, 7500; collision energy, 10/30/60 in the NCE mode; spray voltage, 3.6 kV (positive) or −3.2 kV (negative), respectively.

### Statistical analysis

GraphPad Prism 8.0.2 was used for statistical analysis and to process data. The physiological characteristics of mice are expressed as the mean ± standard error of the mean (X ± SEM). One-way analysis of variance was used to compare multiple groups of data, and *p* < 0.05 was considered to be statistically significant. XploreMET (Metabo-Profile Biotechnology, Shanghai, China) was used to process raw metabolomics data, aggregate QC samples to establish a reference database, compare metabolic signals, correct and normalize missing values, and identify metabolites. This was followed by orthogonal partial least squares discriminant analysis (OPLS-DA). The metabolites were considered statistically significant if a variable had variable importance projection value (VIP) > 1 and *p* < 0.05.

## Results

### LC-MS determination of chemical compounds of YT

As shown in Table 1, Compound Discoverer 3.1 was used for retention time correction, peak recognition and peak extraction according to the secondary mass spectrometry information using Thermo mzCloud online database, Thermo mzValut local databases for substance identification. A total of 58 and 69 compounds were identified in positive and negative ion mode. Among them, the content of 24 components was greater than 1%. The representative compounds include atizoram, α,α-trehalose and several phenolic compounds (rutin, quercetin, catechin, etc.).

**TABLE 1:** HPLC-MS/MS identification of YT extract.

No.	Adduct ion	Chemical name	Molecular formula	PubChem CID	RT (min)	Theoretical value	Test value	Content (%)
1	[M + H]^+^	Atizoram	C_18_H_24_N_2_O_3_	9861730	6.568	316.178	316.1785	8.07
2	[M + H]^–^	α,α-Trehalose	C_12_H_22_O_11_	7427	0.834	342.116	342.1160	6.39
3	[M + H]^+^	9-Benzyl-3,9-diazaspiro[5.5]undecan-2-one	C_16_H_22_N_2_O	272012	6.753	258.173	258.173	6.28
4	[M + H]^–^	2-{[5-(2-Furyl)-1,3,4-oxadiazol-2-yl]sulfanyl}-N-{4-[4-(methylsulfonyl)-1-piperazinyl] phenyl}acetamide	C_19_H_21_N_5_O_5_S_2_	2223179	6.676	463.098	463.097	5.71
5	[M + H]^+^	1,4-Phenylenebis(1-piperidinylmethanone)	C_18_H_24_N_2_O_2_	139883	8.066	300.183	300.183	4.91
6	[M + H]^+^	Quercetin-3β-D-glucoside	C_21_H_20_O_12_	5280804	7.009	464.095	464.095	4.90
7	[M + H]^–^	6-Hydroxyluteolin 7-sophoroside	C_27_H_30_O_17_	44258489	6.15	626.148	626.148	4.80
8	[M + H]^–^	Ethyl2-[({[1-(4-methoxyphenyl)-1H-tetrazol-5-yl]sulfanyl}acetyl)amino]-4,5-dimethyl- 3-thiophenecarboxylate	C_19_H_21_N_5_O_4_S_2_	2238453	7.177	447.103	447.102	4.39
9	[M + H]^–^	Ethyl2-[(2-{[4-(2-phenylethyl)-5-{[(2-thienylcarbonyl)amino]methyl}-4H1,2, 4triazol3yl]sulfanyl}propanoyl)amino]-5,6-dihydro-4H-cyclopenta[b]thiophene-3-carboxylate	C_29_H_31_N_5_O_4_S_3_	4233074	6.534	609.153	609.154	2.98
10	[M + H]^+^	Myricetin-3-rutinoside	C_27_H_30_O_17_	21577860	6.422	626.148	626.148	2.96
11	[M + H]^+^	Kaempferol-7-O-glucoside	C_21_ H_20_ O_11_	10095180	7.503	448.100	448.100	2.91
12	[M + H]^–^	D-(-)-Quinic acid	C_7_ H_12_ O_6_	6508	0.825	192.063	192.062	2.86
13	[M + H]^+^	Quercetin	C_15_ H_10_ O_7_	5280343	7.502	302.042	302.042	2.54%
14	[M + H]^+^	Rutin	C_27_ H_30_ O_16_	5280805	7.502	610.153	610.153	2.15
15	[M + H]^–^	Dihydrobenzoic acid pentose	C_12_ H_14_ O_8_	53462251	4.676	286.068	286.068	1.60
16	[M + H]^+^	Isamoltan	C_16_H_22_N_2_O_2_	127404	6.846	274.168	274.168	1.53
17	[M + H]^–^	7-Chloro-1-(3-ethoxy-4-hydroxyphenyl)-2-(2-furylmethyl)-6-methyl-1,2-dihydrochromeno[2,3-c]pyrrole-3,9-dione	C_25_H_20_Cl N O_6_	667325	6.675	465.097	465.098	1.52
18	[M + H]^–^	Catechin	C_15_H_14_O_6_	107957	5.916	290.079	290.079	1.47
19	[M + H]^–^	8-Hydroxy-6-methoxy-2-oxo-2H-chromen-7-ylβ-D-glucopyranoside	C_16_H_18_O_10_	387151092	5.586	370.090	370.089	1.42
20	[M + H]^–^	(2S,3S)-2-(3,4,5-trihydroxyphenyl)-3,4-dihydro-2H-chromene-3,5,7-triol	C_15_H_14_O_7_	10425234	5.054	306.073	306.073	1.32
21	[M + H]^+^	5,7-Dihydroxy-3-[(2S,3R,4S,5R,6R)-3,4,5-trihydroxy-6-(hydroxymethyl)oxan2yl]oxy-2-(3,4,5-trihydroxyphenyl)chromen-4-one	C_21_H_20_O_13_	5491408	6.586	480.090	480.090	1.20
22	[M + H]^–^	1-(4-Chlorobenzyl)-3-[2-(2,3-dihydro-1,4-benzodioxin-6-yl)-2-oxoethyl]-3-hydroxy-1,3-dihydro-2H-indol-2-one	C_25_H_20_Cl N O_5_	2914732	7.184	449.103	449.103	1.19
23	[M + H]^–^	4-{[(4-Sulfamoylbenzoyl)amino]methyl}-1-(2,3,4,6-tetra-O-acetyl-beta-D-glucopyranosyl)-1H-1,2,3-triazole	C_24_H_29_N_5_O_12_S	11974341	6.528	611.153	611.156	1.18
24	[M + H]^–^	Trimethyl3acetyl1,3,5pentanetricarboxylate	C_13_ H_20_ O_7_	2802950	9.655	288.120	288.120	1.03

### YT extract reduced the body weight, food intake and fat indices of HFD-induced hyperlipidemic mice

**FIGURE 1:**
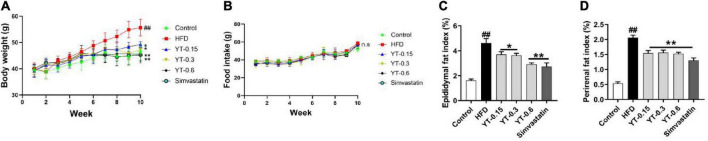
Effect of YT extract on body weight and fat index. **(A)** Body weight, **(B)** food intake, **(C)** epididymal fat index, **(D)** perirenal fat index. Data are presented as mean ± SEM (*n* = 6). ^#^*p* < 0.05 and ^##^*p* < 0.01 vs. the control group; *p < 0.05 and **p < 0.01 vs. the HFD group. ns indicates no significant difference between the control and HFD groups. Fat index = (fat weight/body weight) × 100%.

Figure 1 shows that the body weight, perirenal fat index, and epididymal fat index were significantly increased in mice in the HFD groups compared with those in the control mice (*p* < 0.01). Administration of YT extract resulted in significant decreases in body weight, perirenal fat, and epididymal fat index compared with the HFD group (*p* < 0.05). No significant effects were observed in food intake regulation.

### Effect of YT extract on serum and liver biochemical indices

**FIGURE 2:**
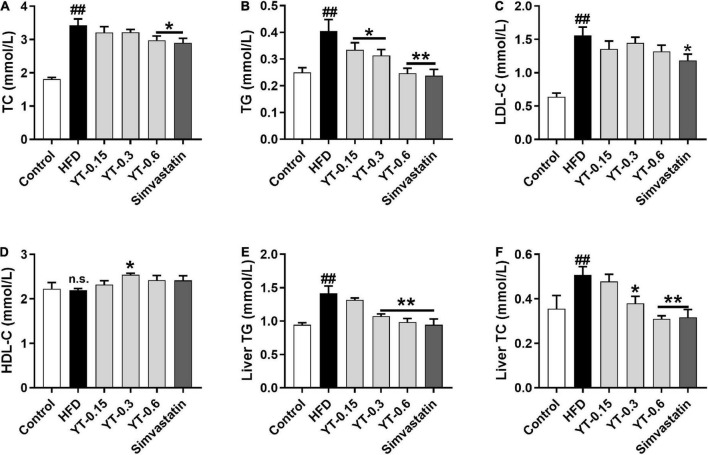
Effect of YT extract on serum and liver biochemical indices. **(A)** TG, **(B)** TC, **(C)** LDL-C, **(D)** HDL-C, **(E)** liver TG, **(F)** liver TC. Data are presented as mean ± SEM (*n* = 6). ^#^*p* < 0.05 and ^##^*p* < 0.01 vs. the control group; *p < 0.05 and **p < 0.01 vs. the HFD group; n.s. indicates no significant difference between the control and HFD groups.

As shown in Figure 2, the serum and liver TC and TG levels of mice in the HFD group were significantly higher (*p* < 0.01) compared with those in the control group. The TC and TG levels in the serum and liver of mice after the administration of three different doses of YT extract or simvastatin showed a decrease compared with those in the HFD group. LDL levels of mice in the HFD group were significantly higher than those of control mice, but there was no significant difference between them with respect to HDL levels. LDL and HDL tended to decrease or increase after YT administration, but these changes were not significant.

### Effects of YT extract on the liver, heart, spleen, and kidney indices

As shown in Table 2, the liver index of mice in the HFD group increased (*p* < 0.05) significantly, whereas the spleen, heart, and kidney indices were not significantly different compared with those of mice in the control group. The liver index of mice receiving high-dose YT extract decreased significantly (*p* < 0.05), whereas the spleen, heart, and kidney indices were not significantly different compared with those of mice in the model group.

**TABLE 2:** Effects of YT extract on the liver, heart, spleen, and kidney indices (X ± SEM, *n* = 6).

Groups	Liver index	Heart index	Spleen index	Kidney index
Control	3.28 ± 0.06	0.42 ± 0.01	0.21 ± 0.01	1.31 ± 0.02
HFD	3.42 ± 0.06#	0.43 ± 0.01	0.22 ± 0.01	1.38 ± 0.03
YT-0.15	3.22 ± 0.06	0.41 ± 0.01	0.18 ± 0.01	1.33 ± 0.02
YT-0.3	3.24 ± 0.09	0.40 ± 0.01	0.18 ± 0.01	1.31 ± 0.03
YT-0.6	3.21 ± 0.06*	0.40 ± 0.01	0.18 ± 0.01	1.28 ± 0.02
Simvastatin	2.98 ± 0.17**	0.41 ± 0.02	0.16 ± 0.01	1.31 ± 0.04

Data are presented as mean ± SEM (*n* = 6). ^#^*p* < 0.05 vs. the control group. *p < 0.05 and **p < 0.01 vs. the HFD group.

### Effect of YT extract on inflammation and oxidative stress

**FIGURE 3:**
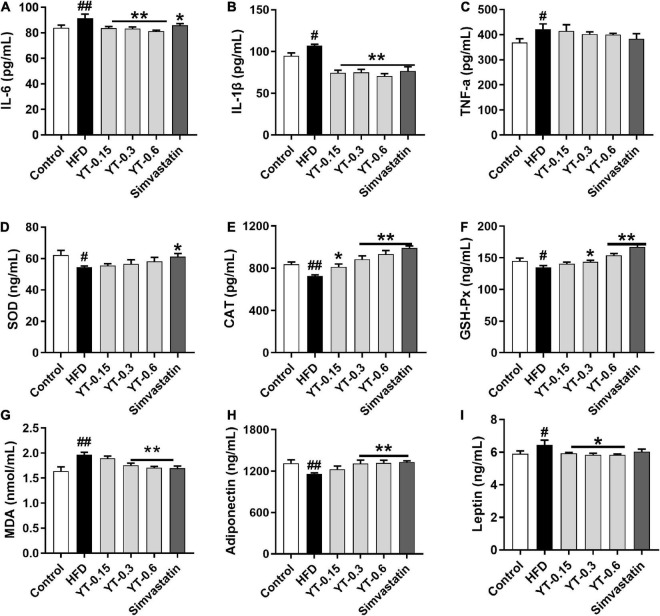
Effect of YT extract on inflammation and oxidative stress. **(A)** IL-6, **(B)** IL-1β, **(C)** TNF-α, **(D)** SOD, **(E)** CAT, **(F)** GSH-Px, **(G)** MDA, **(H)** Adiponectin, **(I)** Leptin. Data are presented as mean ± SEM (*n* = 6). ^#^*p* < 0.05 and ^##^*p* < 0.01 vs. the control group; *p < 0.05 and **p < 0.01 vs. the HFD group.

As shown in Figure 3, compared with the control group, the levels of inflammatory factors IL-6, IL-1β, and TNF-α in the HFD group increased significantly (*p* < 0.05), whereas those of IL-6 and IL-1β decreased significantly after YT administration (*p* < 0.01); TNF-α levels showed a decreasing trend, but the change was not significant (Figures 3A–C). SOD, CAT, GSH-Px, and MDA levels in the HFD group were significantly different (*p* < 0.05) compared with those in the control group. CAT, GSH-Px, and MDA levels in the YT extract and simvastatin groups were significantly different (*p* < 0.01) compared with those in the model group. SOD levels showed an increasing trend, but the increase was not significant (Figures 3D–G). Adiponectin levels of mice in the HFD group decreased significantly (*p* < 0.01) and leptin levels increased significantly (*p* < 0.05) compared with those of mice in the control group. After the administration of YT extract, adiponectin levels in the YT-0.3 g/kg, YT-0.6 g/kg, and simvastatin groups increased significantly (*p* < 0.01), and leptin levels decreased significantly (*p* < 0.05) compared with the model group (Figures 3H, I).

### Pathological changes in the liver and adipose tissues

**FIGURE 4:**
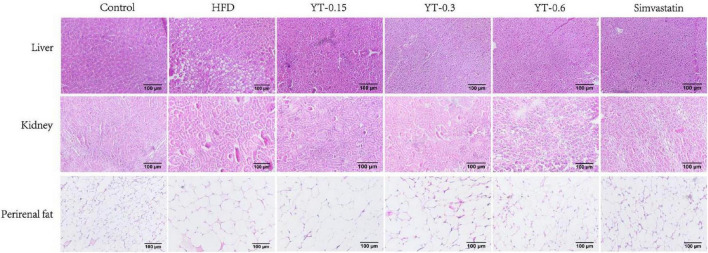
Histological changes in the liver, kidneys, and perirenal fat of six experimental groups of mice. H&E-stained liver and fat tissue sections were observed using an optical microscope at ×400 magnification.

As shown in Figure 4, morphological analysis of the liver, kidney, and adipose tissue showed that the structure and size of hepatocytes in the control group were maintained, whereas those in the HFD group were larger, with serious damage to fat cells and the accumulation of lipid droplets in the cytoplasm. The hepatocyte morphology in the YT extract-treated groups was similar to that of the control group. Lipid droplet accumulation was not observed, and the hepatocytes were normal, especially in the YT-0.6 g/kg group. Similar findings were noted for mice in the simvastatin group.

In the control group, the structure of the renal body was complete; the glomeruli and renal sacs were neatly distributed and had a relatively complete structure, and no obvious lesions were seen. In the HFD group, the glomeruli exhibited increased volume, increased basement membrane thickness, and significant adhesion to epithelial cells. Compared with the HFD group, the YT extract-treated groups and the positive group exhibited a reduction in the glomerular volume of hypertrophy of renal tissue in different amplitude and a decrease in the extent of increase of glomerular basement membrane thickness; thus, the renal lesions of hyperlipidemic mice were treated. Renal tissues from the YT-0.6 g/kg and positive groups showed good healing, and the morphology of the kidneys in the YT-0.6 g/kg group was similar to that of the normal kidneys.

Findings related to the adipose tissues were consistent with those of the liver and kidneys. Adipose cells in the normal group were uniform in size, closely arranged, and well demarcated. The volume of adipocytes in the HFD group showed a significant increase, and the size differed. The size of adipose cells decreased significantly after YT treatment, especially in the YT-0.6 g/kg and simvastatin groups.

### Untargeted metabolomics study

**FIGURE 5:**
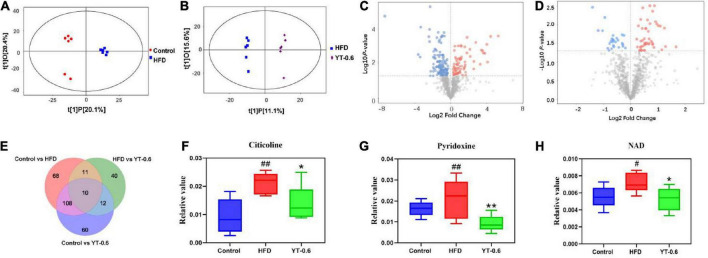
YT extract altered the associated metabolic profiles in HFD-fed mice. **(A)** Orthogonal partial least squares discriminant analysis (OPLS–DA) of the control vs. HFD groups. **(B)** OPLS-DA of HFD-fed vs. the YT-0.6 g/kg group. **(C)** A volcano plot of control vs. HFD groups. **(D)** A volcano plot of HFD-fed vs. the YT-0.6 g/kg group. **(E)** Venn diagram of differential metabolites. **(F)** Three representative differentially expressed metabolites, ^#^*p* < 0.05 and ^##^*p* < 0.01 vs. the control group; *p < 0.05 and **p < 0.01 vs. the HFD group.

**FIGURE 6:**
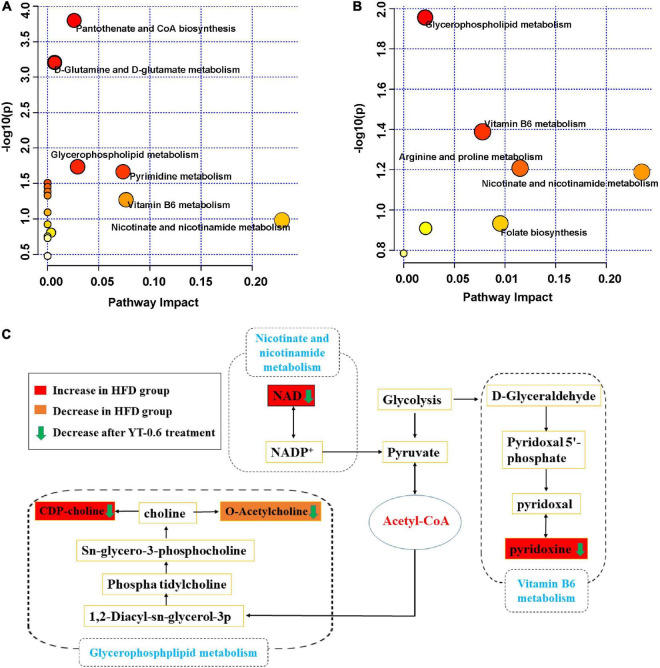
Potential pathways related to the effect of YT extract on HFD diet-induced mice. **(A)** Disturbed metabolic pathways in the control vs. HFD-fed groups. **(B)** Disturbed metabolic pathways in the HFD-fed vs. YT-0.6 g/kg groups. **(C)** Metabolic network of the significantly altered metabolites associated with YT extract intervention.

**FIGURE 7:**
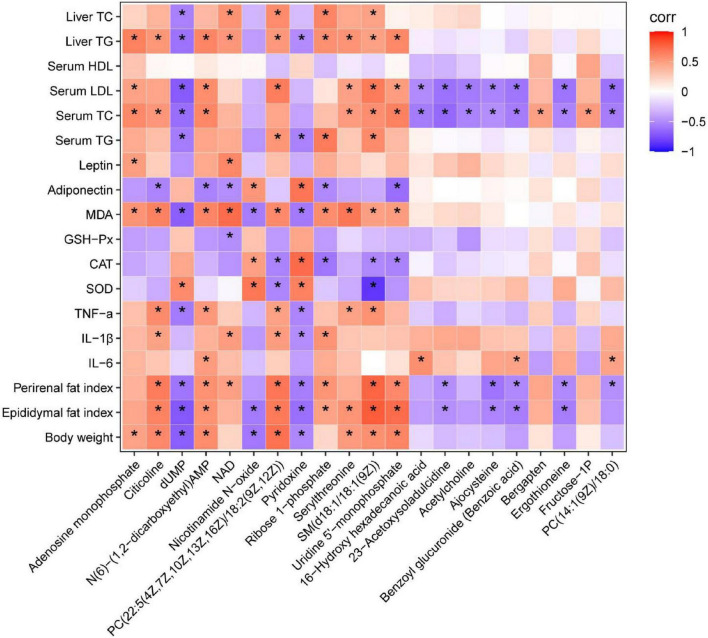
Heatmap analysis of the Spearman’s correlation of significantly changed metabolites and metabolic syndrome-related indices. Positive correlation is in red and negative correlation is in blue. *p < 0.05.

The profound effects of HFD are closely related to complex interactions involving a range of changes in the metabolic profile (37). OPLS-DA can be used to clearly depict changes in the metabolic profile. The OPLS-DA score plot (Figure 5A) shows a significant separation between the control and HFD groups, and (Figure 5B) shows significant separation between the HFD and YT-0.6 g/kg groups. Based on OPLS-DA results, a VIP was generated in each group to determine the contribution of metabolites. Metabolites with VIP > 1 and *p* < 0.05 were considered statistically significant. The enhanced volcano plot shows the differential metabolites selected by multi-criteria assessment between the control and HFD groups (Figure 5C) and between the HFD and YT-0.6 g/kg groups (Figure 5D). A total of 309 differential metabolites were identified from 18 liver samples (Figure 5E). Twenty-one metabolites with significant differences were screened from the HFD and HFD YT-0.6 g/kg groups. Three representative differential metabolites are shown in Figures 5F–H, namely citicoline, serylthreonine, pyridoxine. The results showed that YT-0.6g/kg treatment changed the metabolites of HFD group. After treatment with YT extract, 12 metabolites including adenosine monophosphate, citicoline, dUMP, N(6)-(1,2-dicarboxyethyl)AMP, NAD, nicotinamide N-oxide, PC[22:5(4Z,7Z,10Z,13Z,16Z)/18:2(9Z,12Z)], pyridoxine, ribose-1-phosphate, serylthreonine, SM(d18:1/18:1(9Z)), uridine-5′-monophosphate, and other metabolites returned to their normal trends (Table 3). Metabolic pathway analysis showed that after YT intervention, 10 metabolic pathways, namely, glycerophospholipid metabolism; vitamin B6 metabolism; arginine and proline levels; nicotinate and nicotinamide metabolism; folate biosynthesis; glyoxylate and dicarboxylate metabolism; pentose phosphate pathway; alanine, aspartate, and glutamate metabolism; pyrimidine metabolism; and purine metabolism were changed (Figure 6). Spearman’s correlation analysis showed a correlation between the levels of these metabolites and metabolic disorders, including increased body weight and adipose index, dyslipidemia, inflammation, and oxidative stress (Figure 7).

**TABLE 3:** Differential metabolites were determined by cross-comparison between different groups (control and HFD groups, and HFD and YT-0.6 g/kg groups) using mouse liver samples.

	Metabolites	HFD vs. Control		Trend	*p*-Value	YT-0.6 vs HFD		Trend	*p*-value
		VIP	FC			VIP	FC		
1	Adenosine monophosphate	1.52	0.56	↑	0.015	1.88	1.61	↓	0.032
2	Citicoline	1.59	0.44	↑	0.002	2.04	1.51	↓	0.031
3	dUMP	1.88	1.38	↓	0.001	1.76	0.82	↑	0.038
4	N(6)-(1,2-dicarboxyethyl)AMP	1.52	0.21	↑	0.028	1.98	3.20	↓	0.039
5	NAD	1.35	0.77	↑	0.038	1.97	1.36	↓	0.026
6	Nicotinamide N-oxide	1.36	1.22	↓	0.034	1.99	0.85	↑	0.019
7	PC(22:5(4Z,7Z,10Z,13Z,16Z)/ 18:2(9Z,12Z))	1.07	0.42	↑	0.007	2.05	1.89	↓	0.008
8	Pyridoxine	1.71	2.31	↑	0.004	2.19	0.44	↓	0.014
9	Ribose 1-phosphate	1.43	0.60	↑	0.028	2.23	1.84	↓	0.003
10	Serylthreonine	1.33	0.74	↑	0.023	1.65	1.32	↓	0.049
11	SM(d18:1/18:1(9Z))	1.77	0.34	↑	0.0003	1.99	1.43	↓	0.034
12	Uridine 5′-monophosphate	1.65	0.31	↑	0.005	1.94	1.77	↓	0.033
13	16-Hydroxy hexadecanoic acid	1.58	1.79	↓	0.018	2.05	1.35	↓	0.013
14	23-Acetoxysoladulcidine	2.04	4.51	↓	0.004	1.96	1.95	↓	0.010
15	Acetylcholine	1.36	1.47	↓	0.025	1.72	1.33	↓	0.049
16	Ajocysteine	2.16	3.52	↓	0.0003	1.56	1.69	↓	0.027
17	Benzoyl glucuronide (Benzoic acid)	2.12	11.64	↓	0.002	1.89	1.46	↓	0.034
18	Bergapten	1.51	0.50	↑	0.007	1.62	0.74	↑	0.041
19	Ergothioneine	2.07	9.69	↓	0.011	1.85	2.44	↓	0.039
20	Fructose-1P	1.37	0.62	↑	0.036	1.72	0.73	↑	0.026
21	PC(14:1(9Z)/18:0)	1.87	1.81	↓	0.0009	2.35	1.36	↓	0.004

Significant regulation of potential metabolites in the livers of HFD-fed mice are shown in Table 2. “↑/↓” indicate increased or decreased metabolites. FC indicates “fold change.”

## Discussion

Diet plays a key role in regulating cholesterol balance. Consumption of a high-cholesterol diet is a major risk factor for developing hyperlipidemia (38). In this study, hyperlipidemia was induced in mice by feeding HFD for 10 weeks. We investigated the weight-loss and lipid-lowering effects of YT extract in hyperlipidemic mice to determine whether administration of the extract could reverse HFD-induced dyslipidemia and metabolic abnormalities and exert anti-inflammatory and antioxidant effects. Our results showed that YT extract could significantly inhibit the HFD-induced increase in body weight and reduce the perirenal adipose and epididymal adipose indices. Histopathological changes in the liver and adipose tissue pathology and alterations in the organ indices (liver, kidney, spleen, heart) were determined. The liver index of mice in the HFD group was obviously higher than that of mice in the normal group. Different doses of YT extract and simvastatin could reduce the liver index of mice; however, the kidney, spleen, and heart indices of HFD mice did not show a significant change. Histopathological analyses of liver and fat tissues also confirmed the differences in lipid accumulation in the liver and adipose tissues between HFD-fed and normal diet-fed mice. Lipid droplets in the liver and adipose tissue reduced significantly after treatment with YT extract or simvastatin, suggesting the role of YT extract in lipid metabolism.

Clinically, hyperlipidemia is diagnosed by measuring the four blood lipid indices, which indicate elevated TC, TG, and LDL levels and decreased HDL levels. High blood cholesterol and TG levels are commonly considered the leading biomarkers of hyperlipidemic processes (39). Our findings showed that treatment with YT extract or simvastatin restored lipid balance compared with that in the HFD group, significantly reduced serum and liver TC and TG levels, and showed an improvement in the trends of LDL and HDL levels (40, 41).

SOD, GSH-Px, and CAT are antioxidant enzymes that scavenge reactive oxygen species *in vivo*. Together with MDA, a product of lipid peroxidation, the antioxidant capacity of the body can be determined. We found that SOD, CAT, and GSH-Px activities in the liver of mice in the HFD group decreased significantly, lipid oxidation in the liver was aggravated, and MDA activity was significantly increased. YT extract and simvastatin could increase the activities of SOD, CAT, and GSH-Px, and decrease MDA levels. Moreover, YT extract could inhibit lipid peroxidation and improve the activity of the antioxidant enzymes (14). Accumulation of liver fat can promote the release of the proinflammatory factors TNF-α, IL-6, and IL-1β, leading to liver inflammation and cell damage. We found that IL-6, IL-1β, and TNF-α levels increased significantly in the HFD group compared with those in the control group, and that their levels decreased after treatment with YT extract (42). Therefore, the effect of YT extract in a hyperlipidemic animal model may be related to its antioxidant effect and reduction in inflammation.

Leptin and adiponectin are mainly secreted by adipose tissue and play an essential role in the development and progression of hyperlipidemia. Elevated leptin levels activate Kupffer cells and stellate cells, commencing inflammatory processes in the liver (43). In contrast, adiponectin exerts anti-inflammatory effects in the liver and improves hepatic and peripheral insulin resistance. It also inhibits the production of proinflammatory cytokines such as TNF-α and IL-6 in the liver by suppressing NF-kB activity (44). We found that leptin levels increased significantly in the HFD group; treatment with YT extract or simvastatin led to a significant decrease in leptin. Adiponectin levels, in contrast to those of leptin, were in agreement with previously published results (44). Studies have shown that YT extract has beneficial effects of alleviating hyperlipidemia by regulating leptin and adiponectin levels in the liver.

Moreover, LC-MS analysis reported here indicated polyphenols are the most abundant compounds in YT extract and it has been well reported with effects on obesity, dyslipidemia, blood pressure and glycemia (45), which making them the likely effectors of the pharmacological actions of the YT extract. The activities related to the presence of atizoram, which was another major compound in YT extract, is known to have several pharmacological effects including antiinflammatory and bronchodilatory (46). However, its hypolipidemic function remains to be explored. TCM has been extensively studied on its beneficial effects for treating dyslipidemia. For example, anthraquinones, a major active component of *Rhubarb*, exerted significant lipid-lowering effects by stimulating intestinal motility and reducing intestinal cholesterol absorption (47). In addition, as the main active component of *Rhizoma Coptidis*, berberine decreases blood triglyceride and cholesterol levels in both humans and laboratory animals (48). Although *Xanthoceras sorbifolium* Bunge is well known for its antineuritic and antitumor effects, our results suggest the tender leaves of *Xanthoceras sorbifolium* Bunge include high percentages of polyphenols and exert antioxidant, anti-inflammation and hypolipidemic effects.

Metabolomics is a widely used high-throughput method to identify biomarkers, reveal metabolic pathways, and elucidate the mechanisms of metabolic diseases (49). In this study, untargeted metabolomics technology was used to analyze liver metabolites and the metabolic pathways of YT, and to explore its mechanism in lowering blood lipids. Results from metabolomics analysis revealed that administration of YT extract altered 10 metabolic pathways, including glycerophospholipid metabolism; vitamin B6 metabolism; arginine and proline levels; nicotinate and nicotinamide metabolism; folate biosynthesis; glyoxylate and dicarboxylate metabolism; pentose phosphate pathway; alanine, aspartate, and glutamate metabolism; pyrimidine metabolism; and purine metabolism. Twenty-one metabolites in the YT extract-treated groups were significantly changed compared with those in the HFD group; the altered metabolite pathways included glycerol phospholipid metabolism, vitamin B6 metabolism, and nicotinate and nicotinamide metabolism, which supported the anti-obesity and lipid-lowering effect of YT extract. Metabolites such as citicholine, acetylcholine, pyridoxine, and NAD have been well reported on the regulation of obesity, dyslipidemia, infection, and oxidative stress (50–53). In the present study, correlation analysis between significantly changed metabolites and metabolic syndrome-related indices further demonstrated that YT extract administration was positively associated with weight loss, alleviation of inflammatory and oxidative stress, and lower hyperlipidemia.

Many studies have shown a positive correlation between hyperlipidemia and perturbation of glycerophospholipid metabolism (54, 55). Glycerophospholipids can be classified as phosphatidylcholine (PC), phosphatidylglycerol (PG) and phosphatidylethanolamine (PE) based on their substituents (56). Lysophosphatidylcholines are the core metabolic intermediates of glycerophospholipid metabolism and are considered independent risk factors in the progression of cardiovascular diseases (57). In our study, in the YT extract-treated groups, PC[22:5(4Z,7Z,10Z,13Z,16Z)/18:2(9Z,12Z)], PC(14:1(9Z)/18:0), and LysoPC (17:0) levels were decreased, and the level of LysoPE (16:0/0:0) was increased. This finding was consistent with those reported previously (58). Treatment with YT extract had an inhibitory effect on the increased lysosomes and led to the inhibition of glycerol phospholipid metabolism. The low level of liver lysosomes resulting from treatment with YT extract helps reduce the inflammatory response. Therefore, YT extract plays a key role in alleviating disorders of glycerol and phospholipid metabolism, thereby reducing inflammation and inhibiting the progression of hyperlipidemia.

Vitamin B6 includes three pyridine derivatives, namely, pyridoxal, pyridoxine, and pyridoxamine. Its biologically active form is pyridoxal 5′-phosphate, and 4-pyridoxic acid is its predominant catabolite. Vitamin B6 is a small organic molecule necessary for normal metabolism *in vivo* and plays an important role in many metabolic pathways. Vitamin B6 deficiency affects physiological functions in animals and humans (59–61). Disturbances in lipid metabolism trigger a chronic inflammatory state and promote the production of proinflammatory factors (62). We found that the excretion of pyridoxal in hyperlipidemic mice was higher than that in normal mice, which was consistent with previous studies on hyperlipidemic mice (63, 64). An improvement in abnormal pyridoxal levels suggests that YT extract may regulate vitamin B6 metabolism during hyperlipidemic conditions.

Nicotinic acid inhibits the activity of lipoprotein lipase and LDL-C source and decreases the release of free fatty acids in adipose tissues. It also decreases TC and TG levels and increases HDL-C levels (65). Nicotinic acid, also known as vitamin B3, is a substrate for the synthesis of nicotinamide adenine dinucleotide. In this study, NAD levels in hyperlipidemic mice were higher than those in the control group and returned to normal after treatment with YT extract. Our findings were consistent with previously reported results (66). NAD levels decreased after YT treatment, suggesting that the lipid-lowering effect of YT extract may be related to niacin and nicotinamide metabolism. Overall, YT extract could significantly reverse the high-fat diet-induced abnormal levels of metabolites pyridoxine, NAD, citicholine and acetylcholine, thus interfering with the signaling pathways of vitamin B6 metabolism, nicotinate and nicotinamide metabolism and glycerophospholipid metabolism, thereby being effective in treating hyperlipidemia (Figure 6C). However, further research is required to confirm the levels of target gene or protein expression that are linked to the changed pathways and to demonstrate how YT extract lowers blood lipids at the molecular level.

## Conclusion

We established a HFD-induced mouse model of hyperlipidemia, and the effect of YT extract on hyperlipidemia in mice was studied using pharmacological evaluation and liver metabolomics. Biochemical, ELISA, histopathological, and metabolomics analyses showed that YT extract had lipid-lowering, anti-inflammation and anti-oxidation effects. Our metabolomics study combined univariate and multivariate statistical analyses to identify 21 potential biomarkers associated with hyperlipidemia to distinguish between the metabolic profiles of different groups. YT extract could significantly reverse the abnormal levels of three metabolite pathways of the potential biomarkers in HFD-induced hyperlipidemic mice. YT extract intervention may affect glycerophospholipid metabolism, vitamin B6 metabolism, and nicotinate and nicotinamide metabolism, thereby being effective in preventing and treating hyperlipidemia. Overall, our findings should be insightful in considering YT as a functional beverage for the alleviation of HFD-induced hyperlipidemia, inflammation and oxidative stress.

## Data availability statement

The original contributions presented in this study are included in the article/supplementary material, further inquiries can be directed to the corresponding authors.

## Ethics statement

The animal study was reviewed and approved by the Institutional Animal Care and Use Committee, Inner Mongolia Minzu University (approval no. NM-LL-2021-06-15-1).

## Author contributions

LB and MF designed and funded the project. NT, LA, EE, RQ, XM, and LF performed the animal experiment and analyzed the data. YL, JZ, and MF analyzed and interpreted the metabolomic data. NT and LA wrote the original draft. LB, GB, and MF revised the manuscript. All authors have read and agreed to the published version of the manuscript.
